# Detection of pathogens in dogs with respiratory disease during winter 2023–2024 using multiplex qPCR/RT-qPCR assays and next-generation sequencing

**DOI:** 10.3389/fvets.2025.1617147

**Published:** 2025-08-19

**Authors:** Côme J. Thieulent, Melissa Laverack, Mariano Carossino, Brittany Cronk, Leonardo Cardia Caserta, Diego G. Diel, Udeni B. R. Balasuriya

**Affiliations:** ^1^Department of Pathobiological Sciences, School of Veterinary Medicine, Louisiana State University, Baton Rouge, LA, United States; ^2^Department of Population Medicine and Diagnostic Sciences, Cornell University College of Veterinary Medicine, Ithaca, NY, United States; ^3^Louisiana Animal Disease Diagnostic Laboratory (LSU Diagnostics), School of Veterinary Medicine, Louisiana State University, Baton Rouge, LA, United States

**Keywords:** canine respiratory pathogens, canine infectious respiratory disease complex, CIRDC, qPCR, RT-qPCR, next-generation sequencing, vientovirus

## Abstract

Canine Infectious Respiratory Disease Complex (CIRDC), caused by a diverse range of viral and bacterial pathogens, is the leading cause of respiratory illness in dogs. In the winter of 2023–2024, the United States experienced a noticeable increase in cases consistent with CIRDC. This study investigated the potential association of emerging pathogens with CIRDC cases. It involved the analysis of 50 clinical specimens collected from CIRDC-suspected dogs from six US states between December 2023 and February 2024. All clinical cases presented with respiratory illness characterized mainly by coughing (78%), nasal and ocular discharges (30%), and sneezing (22%). Specimens were tested for 12 known CIRDC-associated pathogens using a previously described panel of one-step TaqMan^®^ multiplex qPCR/RT-qPCR assays designed to detect eight viral and four bacterial pathogens. Specimens were also subjected to next-generation sequencing (NGS) to confirm qPCR/RT-qPCR results and identify potential emerging pathogens. In this study, 64% of samples tested positive for various canine respiratory pathogens. *Mycoplasma canis* was the most frequently detected agent (*n* = 20), followed by *M. cynos* (*n* = 9), canine respiratory coronavirus (*n* = 3), canine parainfluenza virus (*n* = 3), and *Bordetella bronchiseptica* (*n* = 3). Additionally, canine adenovirus type 2, canine herpesvirus 1, and canine distemper virus were also detected in some samples. NGS also identified canine calicivirus, canine circovirus, and, for the first time, vientovirus in a CIRDC-affected dog. This study indicates that CIRDC cases observed in the winter of 2023–2024 were not associated with the emergence of any new pathogens. The clinical relevance of the detection of vientovirus in a single dog remains unknown.

## 1 Introduction

Canine Infectious Respiratory Disease Complex (CIRDC), commonly known as kennel cough, is a highly prevalent and contagious respiratory condition in the dog population worldwide (1, 2). Outbreaks most commonly occur in settings where dogs are housed or gathered nearby, such as boarding facilities, animal shelters, and dog daycares (3, 4). CIRDC manifests as a multifactorial syndrome characterized by acute respiratory signs, including coughing, nasal or ocular discharge, sneezing, fever, and respiratory distress, that generally lasts up to 2 weeks (1). The disease is primarily transmitted through direct dog-to-dog contact and airborne transmission via respiratory secretions (2). Most infections are self-limiting, with affected dogs typically recovering within a few days to a few weeks. CIRDC is a complex disease involving a wide array of viral and bacterial agents, including canine adenovirus 2 (CAdV-2) (5), canine distemper virus (CDV; *Morbilivirus canis*) (6), canine herpesvirus 1 (CaHV-1; *Varicellovirus canidalpha1*) (7), canine influenza virus (CIV) (8), canine pneumovirus (CnPnV) (9, 10), canine parainfluenza virus (CPiV) (11), canine respiratory coronavirus (CRCoV) (12, 13), *Bordetella bronchiseptica* (14), *Mycoplasma canis* (15), *M. cynos* (16), and *Streptococcus equi zooepidemicus* (17, 18). Additionally, sporadic infections with other canine viruses were also reported, including canine calicivirus, canine circovirus, and canine hepacivirus (19–22). Co-infections and certain host factors can contribute to more severe clinical outcomes (23–26). Vaccines targeting specific pathogens associated with CIRD, such as B. bronchiseptica, CAdV-2, CDV, and CPiV, are available in the United States and are commonly administered to puppies (27). Nevertheless, outbreaks continue to be frequently reported (28, 29).

Accurate aetiologic diagnosis of CIRDC is challenging due to the overlapping clinical presentations among pathogens and the frequent occurrence of asymptomatic carriers. For example, *B. bronchiseptica*, *M. cynos*, CaHV-1, and CRCoV are often found in both healthy and clinically ill dogs (12, 30). As such, their contribution to the clinical signs observed in dogs remains uncertain. Asymptomatic dogs significantly contribute to the spread of infections and illnesses among susceptible dogs and may also serve as a reservoir for pathogens between disease outbreaks. Most pathogens have a short incubation period, typically from a few days up to 2 weeks (2). However, given the similarity in clinical signs induced by these pathogens, diagnosis cannot rely solely on history and clinical signs. Thus, laboratory confirmation is essential to confirm clinical diagnosis by accurate pathogen identification. Molecular diagnostic methods such as polymerase chain reaction (PCR) and quantitative PCR (qPCR or reverse transcriptase-qPCR [RT-qPCR]) are widely used for detecting CIRDC agents (31–33). These assays offer high sensitivity and specificity, enabling the detection of active infections. Multiplex qPCR/RT-qPCR platforms, in particular, allow simultaneous detection of multiple pathogens in clinical specimens, making them well-suited for investigating the complex etiology of CIRDC (26, 34). Furthermore, next-generation sequencing (NGS) has become a rapid and comprehensive technique for identifying and characterizing multiple pathogens, including the detection of novel agents in clinical specimens (35). Therefore, these novel molecular techniques (multiplex qPCR/RT-qPCR and NGS) offer high sensitivity, specificity, and quick turnaround time compared to classical virology and bacterial isolation and characterization methods.

In late 2023, veterinarians in several states across the US reported cases of an unusual respiratory illness in dogs, referred to as atypical canine infectious respiratory disease complex (aCIRDC). These cases raised alarms about the possible emergence of a new pathogen (36–39). Unlike typical infections seen in previous years, affected dogs experienced prolonged and severe respiratory clinical signs. Notably, the animals often tested negative for common pathogens associated with canine infectious respiratory disease (CIRD). The atypical nature of CIRDC was further underscored by its poor response to standard antibiotic treatments and a noticeable rise in mortality rates (40). This study aimed to investigate a potential emerging pathogen associated with CIRDC cases during the winter of 2023–2024. A comprehensive one-step multiplex qPCR/RT-qPCR assay was employed to detect the most common CIRDC pathogens, including CAdV-2, CDV, CaHV-1, CIV, CnPnV, CPiV, CRCoV, *B. bronchiseptica, M. canis, M. cynos*, and *S. equi subsp. zooepidemicus*, and SARS-CoV-2. Furthermore, specimens were tested for the possible presence of novel or emerging pathogens by NGS.

## 2 Materials and methods

### 2.1 Clinical specimens

Between December 2023 and February 2024, a total of 50 clinical specimens (pooled nasal and nasopharyngeal swabs) from dogs suspected of having CIRDC were submitted for routine diagnostic testing to the Louisiana Animal Disease Diagnostic Laboratory (LSU Diagnostics) in Baton Rouge, Louisiana, United States (Table 1). Primary practicing veterinarians submitted all specimens to LSU Diagnostics for routine testing; thus, IACUC approval or owner consent was not required for this study. Only samples collected from dogs with respiratory signs were included in this study, regardless of age, body weight, reproductive status, sex, or breed. Both nasal and oropharyngeal specimens were collected by the primary veterinarian using 15 cm sterile flocked collection swabs (VWR, Radnor, PA). The specimens were pooled in 2 mL of BHI Broth (Hardy Diagnostics, Santa Maria, CA) and shipped overnight at 4°C to the LSU Diagnostics. The diagnostic laboratory provided collection swabs and BHI broth along with instructions on how to collect the specimens for the primary practicing veterinarians to ensure optimal sample collection and consistency. The specimens in BHI Broth were packed in ice, and either hand-delivered or submitted vial overnight courier service to LSU Diagnostics for molecular testing. Upon receipt, the samples were immediately processed for molecular diagnostic testing (i.e., multiplex qPCR/RT-qPCR). The remaining BHI broth from each sample was then aliquoted into 500 μL aliquots and stored at −80°C for further use in NGS analysis. Virus and bacteria isolation were not performed.

**Table 1:** Signalment of the samples included in this study (*n* = 50).

Animal number	Breed	Sex	Age	State (USA)	Date received	Clinical signs (if known, duration in days)	Treatment (started before sample collection)
2318341	Labrador Retriever	F	3 Years	GA	12/01/2023	Coughing (7)	ND
2318397	Lhasa Apso	MC	13 Years	TX	12/04/2023	Coughing	Doxycycline, then trimeprazine with prednisolone
2318470	Doberman Pinscher	MC	4 Years	LA	12/05/2023	Coughing and high respiratory rate, crackles on lung auscultation (13)	ND
2318471	Mini Australian Shepherd	MC	4 Years	TX	12/05/2023	Coughing (44)	Doxycycline, then maropitant citrate and amoxicillin with clavulanate potassium
2318520	German Shepherd	F	2 Months	CO	12/06/2023	Coughing and nasal discharge (2)	ND
2318618	Goldendoodle	MC	1 Year	LA	12/07/2023	Coughing, white foam, lethargy, harsh lung sounds, bronchitis (X-ray) (5–6)	ND
2318777	Boxer	MC	20 Months	LA	12/11/2023	Coughing and nasal discharge (1)	ND
2318794	Terrier mix	M	9 Months	TX	12/12/2023	Coughing (7–10)	ND
2318800	Beagle mix	FS	1 Year	LA	12/12/2023	Coughing and heavy breathing (7)	ND
2318902	Dachshund	MC	12 Years	CO	12/13/2023	Dry cough, sneezing, nasal discharge, fever, lethargy and increase respiratory effort	ND
2318903	Doodle mix	MC	1 Year	CO	12/13/2023	Coughing, lethargy and clear nasal discharge (42)	ND
2318904	Australian Shepherd	F	4 Months	CO	12/13/2023	Coughing/sneezing (2)	ND
2318905	Puggle	MC	1 Year	CO	12/13/2023	Coughing (3)	Doxycycline and hydrocodone
2318906	Shih Tzu Mix	FS	4 Years	TX	12/13/2023	Signs of respiratory infection (no specific signs indicated upon submission)	ND
2319063	Labrador Retriever	FS	10 Years	LA	12/15/2023	Coughing (42)	Doxycycline
2319126	Golden Retriever	MC	2 Years	LA	12/18/2023	Coughing, sneezing, nasal and ocular discharges (42)	Doxycycline
2319134	Black Mouth Cur mix	MC	3 Months	TX	12/18/2023	Coughing and nasal discharge (1)	ND
2319135	Mix	M	3 Years	LA	12/18/2023	Signs of respiratory infection (no specific signs indicated upon submission)	ND
2319218	Giant Schnauzer	MC	4 Years	CO	12/19/2023	Coughing and nasal discharge (30)	ND
2319220	French Bull	M	6 Months	CO	12/19/2023	Coughing, sneezing and episodes of difficulty breathing (7)	Amoxicillin and acide clavulanique
2319305	Mix	FS	5 Years	LA	12/21/2023	Coughing, lethargy (few days)	ND
2319322	Bernese Mountain Dog	F	2 Years	CO	12/21/2023	Coughing and nasal discharge (12)	ND
2319336	Cocker Spaniel	F	Unknown	TX	12/21/2023	Coughing (13)	ND
2319515	Boxer	MC	16 Months	LA	12/29/2023	Coughing, mucoid nasal discharge and fever	ND
2400075	Bernese Mountain Dog	M	2 Years	LA	01/02/2024	Low appetite, nasal discharge, sneezing, depression	Doxycycline and clindamycin
2400135	Pitbull	MC	5 Years	NE	01/03/2024	Signs of respiratory infection (no specific signs indicated upon submission)	ND
2400136	Labrador Retriever	M	6 Years	CO	01/03/2024	Coughing (1)	ND
2400178	Greater Swiss Mountain Dog	FS	3 Years	LA	01/03/2024	Coughing, lethargy (2)	ND
2400265	Labrador mix	MC	2 Years	TX	01/04/2024	Coughing (14)	Doxycycline and prednisone
2400309	Cocker Spaniel	FS	9 Years	CO	01/05/2024	Coughing, sneezing, increase respiratory effort (1)	ND
2400381	Mix	MC	10 Years	GA	01/08/2024	Coughing (2)	ND
2400382	Pitbull	M	1 Year	TX	01/08/2024	Coughing, clear nasal and ocular discharge (7)	ND
2400423	Dachshund	F	2 Months	CO	01/09/2024	Coughing (2)	ND
2400502	Border collie	M	3 Years	LA	01/09/2024	Sneezing, hacking, mucoid nasal discharge (7)	ND
2400833	Poodle mix	M	4 Months	CO	01/17/2024	Nasal discharge, vomiting, decreased appetite (1)	ND
2400897	Staffordshire Bull Terrier mix	MC	10 Years	CO	01/18/2024	Coughing, difficulty breathing, lethargy, weakness (30)	ND
2401225	Cavalier King Charles Spaniel	MC	1 Year	TX	01/23/2024	Coughing and sneezing (3)	ND
2401535	Rhodesian Ridgeback	MC	6 Years	CO	01/29/2024	Coughing, sneezing, vomiting white foam, difficulty breathing	ND
2401536	Bulldog	MC	1 Year	CO	01/29/2024	Coughing, hacking, lethargy (1)	ND
2401770	American Bully	M	7 Months	AR	2/2/2024	Chronic respiratory disease (no specific signs indicated upon submission)	ND
2401814	Pekingese mix	FS	3 Years	TX	2/2/2024	Coughing, sneezing, White/green nasal discharge, gagging, choking (2)	ND
2402033	French Bull	MC	13 Years	LA	2/6/2024	Signs of respiratory infection (no specific signs indicated upon submission)	ND
2402110	German Shepherd	MC	12 Years	CO	2/7/2024	Coughing	ND
2402427	Hound mix	N	3 Years	LA	2/12/2024	Signs of respiratory infection (no specific signs indicated upon submission)	ND
2402508	Boxer	F	5 Months	TX	2/15/2024	Coughing, labored breathing, mucoid nasal and ocular discharge (10)	ND
2402850	Labrador Retriever	MC	14 Years	TX	2/21/2024	Coughing, dry sneezing (5)	ND
2402880-7	Shepherd mix	F	11 Months	LA	2/20/2024	Signs of respiratory infection (no specific signs indicated upon submission)	ND
2402880-5	Shepherd mix	M	11 Months	LA	2/20/2024	Intermittent Coughing, louder breathing (42)	ND
2402880-3	Pitbull mix	M	1 Year	LA	2/20/2024	Coughing	ND
2403404	Bernese Mountain Dog	M	3 Years	LA	2/29/2024	Upper respiratory tract infection (no specific signs indicated upon submission)	ND

F, female; FS; female spayed; M, male; MC, male castrated; ND, Not data available; AR, Arizona; CO, Colorado; GA, Georgia; LA, Louisiana; NE, Nebraska; TX, Texas.

### 2.2 Detection of canine respiratory pathogens using one-step multiplex qPCR/RT-qPCR

Each BHI Broth tube with the swabs was vortexed, spun down, and nucleic acid was extracted using the taco™ mini nucleic acid automatic extraction system (GeneReach, Taichung, Taiwan) following the manufacturer’s recommendations. Nucleic acid was extracted from 100 μL of BHI Broth and then eluted in 100 μL of elution buffer following the manufacturer’s instructions. The extracted nucleic acid from each sample was immediately used in multiplex qPCR/RT-qPCR testing, and the remaining nucleic acid was archived at −80°C. Routine diagnostic testing was conducted using multiplex one-step qPCR/RT-qPCR assays to detect 12 canine respiratory pathogens, including CAdV-2, CDV, CaHV-1, CIV, CPiV, CPnV, CRCoV, SARS-CoV-2, *B. bronchiseptica*, *M. canis*, *M. cynos*, and *S. equi* subsp. *zooepidemicus*, as previously described (26). Briefly, five μl of template nucleic acid per reaction was mixed with the master mix in a final volume of 25 uL reaction and ran on a 7,500 Fast Real-Time PCR System (Applied Biosystems, Waltham, MA) with the following thermal profile: a reverse transcription step (20 min. at 50°C) followed by an initial activation step (15 min. of at 95°C) and 40 cycles of denaturation and annealing/extension (45 sec. at 94°C and 75 s. at 60°C). The cycle threshold (Ct) cut-off for the detection of positive/negative samples was used as previously described (26).

### 2.3 Detection of canine respiratory pathogens using next-generation sequencing

After nucleic acid extraction for real-time RT-qPCR/qPCR, the remaining BHI Broth samples were aliquoted (approximately 500 μL per vial) and stored at −80°C. Approximately 500 μL of the frozen archived samples were shipped on dry ice to the Animal Health and Diagnostic Center (AHDC) at Cornell University, NY, a leading NGS laboratory for NGS as previously described (41). The NGS protocol applied was specifically optimized for virus detection and was used here exclusively for research purposes, rather than routine diagnostic testing. Upon receipt, samples were subjected to an enzymatic cocktail treatment composed of 10 × DNase I buffer, DNase I, Turbo DNase, RNase Cocktail™ (Thermo Fisher Scientific, Waltham, MA), Baseline-ZERO DNase (Lucigen, Middleton, WI), Benzonase (Sigma-Aldrich, Saint-Louis, MO) and RNase ONE™ Ribonuclease (Promega, Madison, WI) to deplete host and bacterial nucleic acids. Additionally, Mengovirus strain vMC0 (Mengo Extraction control kit, bioMérieux, Marcy-l’Étoile, France) was added to each sample at a concentration of 2.17 × 10^6^ copies/ml, serving as an exogenous internal control for the NGS sample preparation, library preparation, and sequencing reactions (42). The enzyme cocktail, Mengovirus, and sample mixture were incubated for 90 min at 37°C. After enzymatic treatment, nucleic acid extraction was performed using the QIAamp^®^ MinElute^®^ Virus Spin Kit (Qiagen, Hilden, Germany). Purified nucleic acids were subjected to sequence-independent, single-primer amplification (SISPA) procedures modified from a previously reported protocol (43). In brief, 11 μL of nucleic acids were used in a reverse transcription reaction with 100 pmol of primer FR20RV-12 N (5′-GCCGGAGCTCTGCAGATATCNNNNNNNNNNNN-3′) using SuperScript™ IV reverse transcriptase (Thermo Fisher Scientific), followed by second-strand synthesis using the Klenow Fragment of DNA polymerase (New England Biolabs, Ipswich, MA) with primer FR20RV-12 N at 10 pmol. After purification using Agencourt AMPure XP beads (Beckman Coulter, Brea, CA), SISPA PCR amplification was conducted with TaKaRa Taq DNA Polymerase (Takara, Kusatsu, Japan) using the primer FR20RV (5′-GCCGGAGCTCTGCAGATATC-3′) at 10 pmol. Sequencing libraries were prepared using 143 ng of double-stranded DNA as input to the SQK-LSK109 kit and barcoded individually using the EXP-NBD196 Native barcodes (Oxford Nanopore Technologies (ONT), Oxford, United Kingdom). The sequencing was performed on the FLO-MIN106 MinION flow cell r9.4.1 using the GridION Sequencer (ONT). High-accuracy base calling was performed by the GridION with the parameters “--require_barcodes_both_ends” and “--detect_mid_strand_barcodes.” Fastq reads were then filtered by size and quality using Chopper (44), host-removed using NanoLyse (45), and classified using Kraken (v2.1.0) (46, 47) followed by relative abundance estimation using Bracken. Bioinformatics data analysis was performed using Base2Bio (Oshkosh, WI).

### 2.4 Detection of vientovirus by standard PCR and phylogenetic analysis

The presence of the vientovirus in original clinical sample was confirmed by two distinct PCR assays targeting capsid (Cap) and the replicative (Rep) open reading frames in a total volume of 25 μL containing 12.5 μL of AccuStart II PCR ToughMix (2X) (Quantabio, Beverly, MS), 2.5 μL of a forward and reverse primer mix (5 μM; F_Cap: GATATGCATCAAGAAAGAGAGTTTATCG; R_Cap: ATTCTTAACACCTTTGCCAGAAATC; F_Rev: CCGTCTAGTAATCTGAGGAGGA; R_Rev: TGTGGTTTCATGGAGATACAGG), 5 μL of nuclease-free water and 5 μL of DNA template. Thermal cycling was performed on a Mastercycler^®^ × 50 – PCR Thermocycler (Eppendorf, Hamburg, Germany) using the following conditions: an initial denaturation step (3 min. at 94°C) and 40 cycles of denaturation, annealing, and extension (30 s. at 94°C, 30 s. at 60°C, 30 s. at 70°C). The PCR products were analyzed by gel electrophoresis on a 1% agarose gel (ThermoFisher Scientific, Waltham, MA), and the expected band sizes were as follows: Cap: 200 bp; Rev.: 218 bp. The PCR products were submitted for Sanger sequencing to Eurofins Genomics (Louisville, KY).

## 3 Results

### 3.1 Clinical history

A total of 50 pooled nasal and oropharyngeal swab samples from dogs with respiratory illness were submitted to LSU Diagnostic Laboratory—24 in December 2023, 15 in January 2024, and 11 in February 2024 (Table 1). Out of the 50 samples, 35 were collected from male (70%) and 15 were collected from female (30%) dogs. The age ranged from 2 months to 13 years (average = 3.8 years). Samples were received from six states, including Arizona (AR; *n* = 1), Colorado (CO; *n* = 16), Georgia (GA; *n* = 2), Louisiana (LA; *n* = 18), Nebraska (NE; *n* = 1), and Texas (TX; *n* = 12). Among the clinical signs reported by the veterinarians, coughing was the most prevalent (*n* = 39; 78%), followed by nasal and ocular discharges (*n* = 15; 30%), sneezing (*n* = 11; 22%), lethargy (*n* = 7; 14%) and high respiratory rate/heavy breathing (*n* = 7; 14%). Other clinical signs, including fever (*n* = 2; 4%), vomiting (*n* = 2; 4%), decreased appetite (*n* = 2; 4%), choking (*n* = 2; 4%), hacking (*n* = 2; 4%) and weakness (*n* = 1; 2%) were also reported. For six of the dogs, signs of respiratory infection were indicated, but no specific signs were provided in the submission forms. Clinical signs were reported up to 44 days before specimens were collected (Interquartile range [IQR] = 11).

### 3.2 Pathogen identification using multiplex qPCR/RT-qPCR assays

Thirty samples (60%) were positive for at least one of the tested pathogens, while twenty samples (40%) were negative (Table 2). Eight different pathogens were detected, including *M. canis* (*n* = 20; 40%), *M. cynos* (*n* = 9; 18%), CRCoV (*n* = 3; 6%), CPiV (*n* = 3; 6%), *B. bronchiseptica* (*n* = 3; 6%), CAdV-2 (*n* = 1; 2%), CaHV-1 (*n* = 1; 2%) and CDV (*n* = 1; 2%). Eight samples were positive for more than one pathogen, including seven positive samples for two pathogens (CRCoV and *M. canis* [*n* = 2], CPiV and *M. canis* [*n* = 2], *M. cynos* and *M. canis* [*n* = 3]) and one sample positive for five pathogens (CDV, CRCoV, CaHV-1, *B. bronchiseptica,* and *M. canis*). *M. canis* was detected in all the samples where more than a single pathogen was identified.

**Table 2:** Detection of viral and bacterial agents in samples collected from dogs with respiratory disorders using NGS and qPCR/RT-qPCR.

Animal number	AHDC NGS viral^a^	AHDC NGS bacterial^b^	LADDL qPCR/RT-qPCR
2318341	ND	ND	*M. canis*
2318520	Canine calicivirus (0.98)	*M. canis* (0.28)	*M. canis*
2318618	ND	*M. canis* (0.37)	*M. canis*
2318777	Canine calicivirus (1.31)	ND	*M. canis*
2318800	ND	ND	*M. canis*
2318904	ND	*M. canis* (0.016)	*M. canis*
2318906	ND	*M. canis* (0.06)	*M. canis*
2319063	ND	ND	*B. bronchiseptica*
2319126	ND	*M. canis* (0.36)	*M. cynos*
2319134	ND	*M. cynos* (0.05)	*M. cynos*
2319220	ND	*M. canis* (1.50) *M. cynos* (0.32)	*M. canis*
2319322	ND	*M. canis* (0.19)	*M. canis*
2319336	ND	*M. canis* (0.56)	*M. canis*
2319515	CRCoV (9.25)	*M. canis* (0.05)	CRCoV *M. canis*
2400075	ND	*M. cynos* (4.2)	*M. cynos*
2400136	CPiV (0.47)	ND	CPiV
2400178	ND	ND	CAdV-2
2400382	CRCoV (2.89)	ND	CRCoV *M. canis*
2400423	CPiV (0.02)	*M. canis* (0.35)	CPiV *M. canis*
2400502	ND	ND	*B. bronchispetica*
2400833	ND	*M. cynos* (8.1)	*M. canis* *M. cynos*
2400897	Human oral-associated vientovirus (4.52)	ND	ND
2401225	ND	*M. canis* (0.69)	*M. canis*
2401536	Canine circovirus (0.08)	ND	*M. canis*
2401770	CRCoV (17.99) CaHV-1 (2.46) CDV (0.37)	*B. bronchiseptica* (7.23) *M. canis* (2.1)	CRCoV CaHV-1 CDV *B. bronchiseptica* *M. canis*
2401814	ND	*M. canis* (0.8) *M. cynos* (0.2)	*M. canis* *M. cynos*
2402427	CPiV (8.47)	*M. canis* (0.14)	CPiV *M. canis*
2402508	Canine circovirus (0.16)	*M. canis* (0.03) *M. cynos* (24.00)	*M. canis* *M. cynos*
2402850	ND	*M. canis* (0.006)	*M. canis*
2402880-7	ND	*M. cynos* (0.002)	*M. cynos*
2402880-5	ND	*M. cynos* (0.95)	*M. cynos*
2402880-3	ND	*M. cynos* (1.2)	*M. cynos*

Specimens collected from animals 2,318,397, 2,318,470, 2,318,471, 2,318,794, 2,318,902, 2,318,903, 2,318,905, 2,319,135, 2,319,218, 2,319,305, 2,400,135, 2,400,265, 2,400,309, 2,400,381, 2,401,535, 2,402,033, 2,402,110, and 243,404 were negative for viral and bacterial agents related to respiratory infection by NGS and qPCR/RT-qPCR. AHDC, Animal Health and Diagnostic Center, Cornell University; LADDL, Louisiana Animal Disease Diagnostic Laboratory, Louisiana State University; ND, Not detected; NGS, Next Generation Sequencing.

a Percentage of reads classified.

b Relative abundance.

### 3.3 Pathogen identification using next-generation sequencing

NGS results matched those of qPCR/RT-qPCR in forty-one samples (82%), while discrepancies were noted in nine samples (18%) (Table 2). Among these, one sample was positive for *M. cynos* by qPCR, but *M. canis* was identified by NGS, and another sample was positive for *M. canis* by qPCR, while both *M. canis* and *M. cynos* were identified by NGS. In the remaining seven specimens with discrepancies, NGS did not detect *B. bronchiseptica*, *M. canis*, and CAdV-2, while qPCR/RT-qPCR did. Importantly, NGS identified canine calicivirus (*n* = 2) and canine circovirus (*n* = 2), two viruses that were not included in the multiplex qPCR/RT-PCR panel. Both canine calicivirus-positive samples and one of the canine circovirus-positive samples were also positive for *M. canis*, while the second circovirus-positive sample tested positive for *M. cynos*. Additionally, vientovirus was identified in one sample collected from a dog that presented with coughing, difficulty breathing, lethargy, and weakness for 30 days, with the sample tested being collected at that timepoint. No other pathogens were detected in this specimen. The specimens that yielded contradicting results with multiplex qPCR/RT-qPCR versus NGS were retested to confirm the validity of the test results. As expected, the results remained unchanged in repeat testing and NGS. Finally, in 18 (36%) of the samples, no pathogens were detected by either multiplex real-time qPCR/RT-qPCR or by NGS.

### 3.4 Confirmation of vientovirus DNA by standard PCR and phylogenetic analysis of capsid and replicative genes

To confirm the presence of vientovirus DNA in sample 2,400,897, two specific PCR assays targeting the capsid (Cap) and replicative (Rep) genes were developed based on the full-genome sequence obtained by NGS and the sequence was deposited in GenBank (Vientovirus/USA/CO/2024/2400897; GenBank accession #: PQ450187). These assays detected vientovirus in the original specimen but not in any other samples (Supplementary Figure 1A) and were further validated by Sanger sequencing. Phylogenetic analysis showed high similarity to known vientovirus isolates (Supplementary Figures 1B,C). To rule out human DNA contamination, species-level classification of 500 raw reads revealed that over 60% aligned with *Canis lupus familiaris*, 0.51% corresponded to the human oral-associated vientovirus AV, and only 0.25% mapped to human DNA (Supplementary Figure 1D).

## 4 Discussion

CIRDC is a complex infectious disease in dogs, attributable to single or synergistic actions of diverse viral and bacterial agents (1, 23). A rapid detection of the implicated pathogens is important to provide the most appropriate treatment strategy and implement proper biosecurity measures to prevent the spread of respiratory infections. Moreover, the detection of emerging pathogens is essential to understand their epidemiology and to inform owners and public health officials. An increase in the number of CIRDC cases in the US was perceived in late 2023 and was relayed by several public news outlets (36–39). Between December 2023 and February 2024, 50 nasal and nasopharyngeal swab specimens were collected from dogs experiencing respiratory disease and submitted for diagnostic testing to LSU Diagnostics. These samples were tested for common canine respiratory pathogens by multiplex qPCR/RT-qPCR and by NGS to attempt the identification of potential emerging pathogens.

When considering both qPCR/RT-qPCR and NGS results, 32 samples (64%) were positive for one pathogen known or suspected to induce respiratory infections in dogs. *M. canis* and *M. cynos* are the most common pathogens identified from CIRDC dogs, which is consistent with previous findings (24, 26, 34). Additionally, cases of CRCoV, CPiV, and *B. bronchiseptica* were reported, aligning with earlier reports (23, 48). The use of NGS has the advantage of allowing the identification of pathogens not included in the qPCR/RT-qPCR diagnostic panels. Here, canine calicivirus, canine circovirus and, for the first time, vientovirus (family: *Redondoviridae*) were detected in dogs with CIRDC. Canine calicivirus (CaCV) is an unclassified virus in the family *Caliciviridae*. CaCV was isolated and characterized for the first time in 1985 from the feces of a diarrheic dog in the US (49). Clinical signs induced by CaCV are diverse and range from the absence of clinical signs to severe diseases characterized by bloody diarrhea, hemorrhagic gastroenteritis, vomiting, and depression (20). However, CaCV has not been detected in respiratory tract specimens before. Additionally, experimentally infected dogs with different strains of CaCV failed to induce respiratory clinical signs (20, 49). Canine circovirus (species: *Circovirus canine*; genus: *Circovirus*; family: *Circoviridae*) was detected for the first time in 2011 in the serum of healthy dogs (50). The pathogenicity of canine circovirus is currently poorly understood, with the virus being mainly detected in fecal samples. Canine circovirus infection has been associated with clinical profiles of acute gastroenteritis, hemorrhagic diarrhea, signs of vasculitis, lymphadenitis, thrombocytopenia, neutropenia, and lymphopenia (51). Canine circovirus has also been associated with respiratory signs in dogs such as dyspnea, nasal discharge, cough, or rales (52). However, the virus has also been detected in samples of asymptomatic dogs, so its role in clinical disease and its pathogenesis is not clearly defined. It is interesting to note that *M. canis*, which is commonly found in the respiratory tract of healthy dogs, was detected in all samples that tested positive for CaCV and canine circovirus. Although it is not yet clear whether *M. canis* contributes to secondary infections in the upper airways, it is generally considered a commensal or opportunistic cofactor in respiratory and urogenital tract diseases in dogs (15). In this study, vientovirus DNA was found in a nasal/pharyngeal swab from one dog with prolonged respiratory illness (30 days) with no other detectable pathogens. Vientovirus (*Torbevirus viento*), a member of the *Redondoviridae* family (53), has so far been identified only in human respiratory samples through metagenomics, with no confirmed detection in animals (such as pigs, chickens, ducks, and dogs) or the environment (freshwater, marine environments, or soil) (54, 55). The transmission of this virus from humans to animals or animals to humans has not yet been demonstrated. Although prior infection with common respiratory agents cannot be ruled out for the dog positive for vientovirus DNA, the relevance of this virus to the clinical signs remains uncertain. Both NGS and PCR confirmed its detection. However, sample collection occurred late in the illness, and no other samples were found to be positive. While human DNA contamination was considered, it is unlikely due to the low human read counts and the inability to detect this virus in other specimens, simultaneously processed and tested by the same technicians. However, the detected viral sequence showed greater than 90% similarity to human-associated strains. Without supporting pathological or serological data, the role of the vientovirus virus in canine disease remains unclear.

Our results indicate that dogs from different states were infected with several known canine pathogens during the winter of 2023–2024, originating from six states in the US. Previously, studies have focused on detecting the nasal virome in dogs, which identified similar pathogens, including a novel *Taupapillomavirus* (CPV21–23) (56). Similarly, studies have focused on identifying and comparing the canine microbiome, which has revealed numerous bacterial taxa (57). It is important to highlight that the NGS protocol employed in this study was specifically optimized for viral detection, showing concordant results with the multiplex qPCR/RT-qPCR. Although the NGS protocol was not optimized for bacterial identification, it was applied for this purpose, which may partly explain the differences observed between the two methods in detecting bacterial pathogens, particularly the reduced bacterial detection by NGS. The nucleic acid extraction method, which includes a step to deplete host nucleic acids, may have contributed to the failure to detect certain bacteria. While our findings highlight several pathogens detected during the outbreak, it is important to consider factors that may influence diagnostic test results and the interpretation of negative findings. The quality of test results depends on the timely collection of appropriate samples during the infection. A negative qPCR/RT-qPCR or NGS test result means that the target virus or bacteria were not detected in the specimen. This could be due to the sample being collected too early or too late in the course of infection, resulting in insufficient target DNA or RNA for detection, or because no infection was present. In this study, 38% of the samples yielded negative results. The absence of pathogen identification in these cases could be attributed to several factors, including, but not limited to, lower respiratory tract infections, nucleic acids depletion techniques used during samples preparation for NGS (i.e., specific for bacteria detection), pathogen clearance before sample collection, antibiotic treatment by veterinarians, or non-infectious causes that the attending veterinarians may have considered. Based on available information, antibiotic treatment had been initiated in eight dogs before sample collection, which may have influenced the results by reducing the detectability of primary bacterial pathogens at the time of sampling. Follow-up was not possible for these cases, which limited the ability to gather additional clinical data or repeat testing to clarify the etiology of illness. Further investigation into the other respiratory causes of these negative cases could provide valuable insights and may be an important area for future research. Given the limited number of samples available during the Winter 2023–2024 outbreak (*n* = 50), it is essential to note that the relatively small sample size does not represent the overall US canine population, and the findings of this study should be considered exploratory. Nevertheless, the results provide valuable preliminary insights that may inform future, larger-scale investigations into CIRDC-associated pathogens. Additionally, the vaccine status of these animals was unknown. For these reasons, the association of other known or new respiratory pathogens in CIRDC cases across the country cannot be ruled out. Finally, nasal and pharyngeal swabs were pooled together before performing the diagnostic assays; therefore, the comparison of the two collection methods was not possible in this study. The SISPA method is a valuable tool for viral and bacterial metagenomic studies (58–60); however, like other NGS methods, it has limitations that affect its sensitivity. A key challenge is the requirement for high viral loads for adequate amplification, as lower concentrations lead to non-specific products and poor genome recovery. SISPA is also prone to amplification biases, frequently resulting in incomplete viral genome assemblies, particularly when viral titers are low (61). Additionally, background contamination from host nucleic acids can reduce sensitivity, necessitating stringent preprocessing and enrichment strategies.

While *Mycoplasma* species were detected at a high rate in these samples, we believe it is unlikely that they were the primary pathogen responsible for the outbreak. Instead, it may have acted as a secondary pathogen, contributing to the overall disease burden. Future studies focusing on viral and bacterial co-infections, as well as the timing of pathogen detection relative to clinical signs, would be valuable in further elucidating the role of *Mycoplasma* in CIRDC.

In summary, our results suggest that the increase in CIRDC cases observed in the winter of 2023–2024 is unlikely to be attributed to a single pathogen or the emergence of a novel viral or bacterial pathogen. No conclusive evidence of a new causative agent was identified in this study.

## Data Availability

The original contributions presented in the study are included in the article/Supplementary material, further inquiries can be directed to the corresponding author.
