# Supplementary material for: Detection of pathogens in dogs with respiratory disease during winter 2023–2024 using multiplex qPCR/RT-qPCR assays and next-generation sequencing
Source: Front Vet Sci. 2025 Aug 19;12:1617147. doi: 10.3389/fvets.2025.1617147 (PMC12402941; doi:10.3389/fvets.2025.1617147)
Supplement: Supplementary file 1 [file Image_1.pdf]

Supplementary Material

1 Supplementary Figures and Tables

1.1 Supplementary Figure

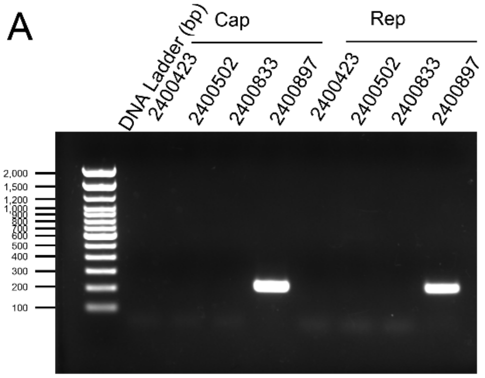

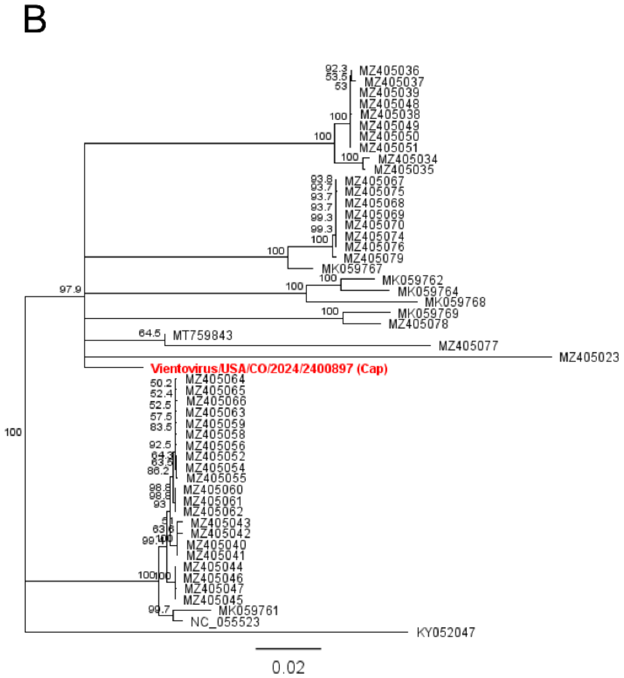

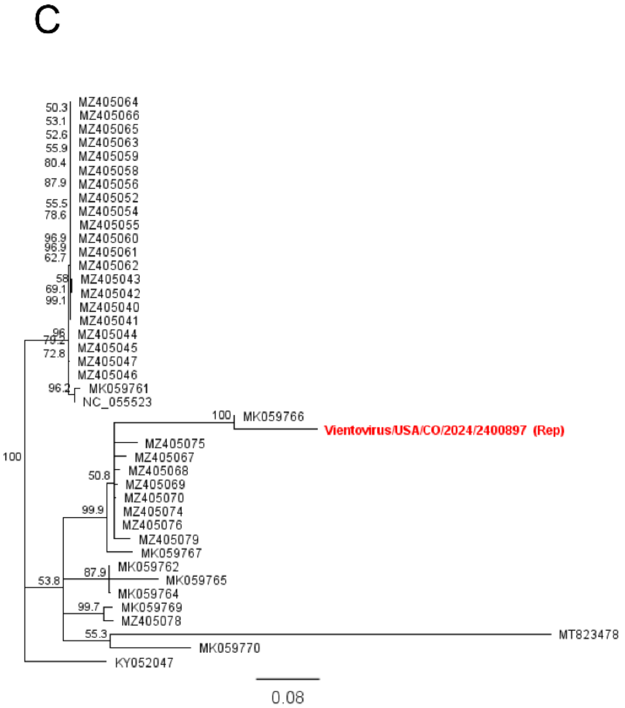

D

Species	% Reads
Canis lupus familiaris	**60.051**
Canis lupus	12.468
Mengo virus	11.196
Lutra lutra	2.29
Treponema denticola ATCC 35405	1.527
Mycoplasma feliminutum	1.527
Desulfomicrobium orale DSM 12838	1.272
Human respiratory circular DNA virus	1.018
Capnocytophaga canimorsus	0.763
Meles meles	0.763
Treponema putidum	0.763
Felis catus	0.509
Desulfobulbus oralis	0.509
Streptococcus porcinus	0.509
uncultured bacterium gwf2_scaffold_210	0.509
**Human oral-associated vientovirus AV**	**0.509**
Fusobacterium canifelinum	0.509
Lachnospiraceae bacterium oral taxon 500	0.254
Treponema succinifaciens DSM 2489	0.254
Kosakonia sp. MUSA4	0.254
Circoviridae sp.	0.254
Cervus elaphus	0.254
Fretibacterium fastidiosum	0.254
Parvimonas micra	0.254
Ottowia oryzae	0.254
Acholeplasmatales bacterium feline oral taxon 303	0.254
Helicobacter cinaedi	0.254
Helicobacter hepaticus	0.254
Sulfurospirillum barnesii	0.254
Homo sapiens	**0.254**

Supplementary Figure 1: Detection of Vientovirus by conventional PCR and phylogenetic analysis. (**A**) Gel electrophoresis showing the amplification of the vientovirus capsid (Cap) and replicative (Rep) genes by conventional PCR in sample 2400897. Band sizes (200 bp and 218 bp) are specific to the Cap and Rep amplicons, respectively. Phylogenetic analysis of the vientovirus strains based on the capsid (Cap) sequences (**B**) and the replicative (Rep) sequence (**C**). Genome alignment and the phylogenetic tree were constructed using Geneious 6.1.8 software. Alignment was performed using the Geneious alignment algorithm, the trees were generated using the neighbor-joining method, and the Tamura-Nei model was used for genetic distance estimation. Bootstrapping with 1,000 replicates was performed to assess the robustness of the tree. The phylogenetic trees included the vientovirus strain obtained from this investigation (Vientovirus/USA/CO/2024/2400897: represented in red), along with other vientovirus strains available from GenBank. The reference distance bar and bootstrap values are indicated. (**D**) Table showing the results from the fastq_species_detector program, which performs BLAST on a subset of 500 raw reads and provides the identified species along with the corresponding percentage of aligned reads.
